# Central Retinal Artery Occlusion in a Young Patient With a Hidden Unusual Sickle Cell Trait

**DOI:** 10.7759/cureus.34865

**Published:** 2023-02-11

**Authors:** Valmore A Semidey, Moustafa S Magliyah, Naif Alali, Faris Hashem, Hani B ALBalawi

**Affiliations:** 1 Vitreoretinal Division, King Khaled Eye Specialist Hospital, Riyadh, SAU; 2 Department of Ophthalmology, Prince Mohammed Medical City, Aljouf, SAU; 3 Ophthalmology Division, Department of Surgery, Faculty of Medicine, University of Tabuk, Tabuk, SAU

**Keywords:** vision loss, hemoglobin electrophoresis, sickle cell trait, crao, central retinal artery occlusion

## Abstract

Sickle cell trait is considered a benign condition. Ophthalmic manifestations are infrequent but can result in significant visual deterioration. We present a case of a 33-year-old male, not known to have any medical illnesses, who presented to the ophthalmological emergency room complaining of a sudden onset of painless and profound left eye vision loss for 12 hours. The patient denied any medication use, past eye trauma, or surgery. On detailed ophthalmologic examination, the best-corrected visual acuity (BCVA) was 20/20 in the right eye and hand movement in the left eye. Dilated fundus examination of the left eye showed a central retinal artery occlusion (CRAO) with pale, white retinal swelling and a macular cherry-red spot. Fundus fluorescein angiography showed delayed arterial filling with persistently reduced macular perfusion. CRAO was diagnosed in an otherwise healthy young male. Systemic workup was negative except for protein electrophoresis, which showed sickle cell trait, and HbA1C was 7.8%. Later, atrophic macular changes with a pale optic disc were observed, and BCVA was reduced to light perception. CRAO in young patients amounts to diverse causes, which require extensive systemic workup. In addition, the concurrence of the sickle cell trait with diabetes mellitus might have a role in CRAO development.

## Introduction

In 1859, von Graefe first described central retinal artery occlusion (CRAO) [1]. It is considered an ocular emergency and an ocular analog of acute ischemic cerebral stroke. Management includes controlling the acute event, localizing the site of vascular occlusion, and trying to prevent other occurrences in the eye or other locations [2]. CRAO is characterized by a compromised blood flow to the inner layers of the retina, which are supplied by the central retinal artery. It presents slightly more in men than women [3]. The incidence is around one in 100,000 in the general population [4]. Based on pathogenesis, CRAO broadly can be classified as arteritic and non-arteritic. Non-arteritic presentation is responsible for more than 90% of the CRAO cases, and 70% of non-arteritic cases are associated with carotid artery disease; it is worth mentioning that in the EAGLE study, more than 70% of patients had carotid artery stenosis [5]. Other non-arteritic causes include hematological conditions and hypercoagulable states (such as antiphospholipid syndrome, deficiency of factor V Leiden, deficiency of antithrombin, protein C or protein S deficiency, elevated levels of fibrinogen or dysfunctional fibrinogen, elevated levels of coagulation factors VIII, IX, or XI, aplastic anemia, erythrocytosis, hemochromatosis, immune thrombocytopenic purpura, leukemia, and sickle cell disease), emboli of cardiac origin, and others. Giant cell arteritis is the most common etiology of the arteritic type of CRAO [6]. Patients with CRAO usually complain of unilateral, sudden, painless, and profound loss of vision, and around one-third of patients have visual acuity of counting fingers or worse [7]. In young patients, CRAO may be correlated to a known preexisting systemic disease, the initial presentation of an undiagnosed disease, or hereditary diseases such as Fabry disease. Those patients usually have more obscure and diverse etiological causes that require extensive workup, as in our case [8]. We report a rare case of CRAO in a young male who presented to the ophthalmology emergency room complaining of sudden painless loss of vision, claiming that he is medically free.

## Case presentation

**Figure 1:**
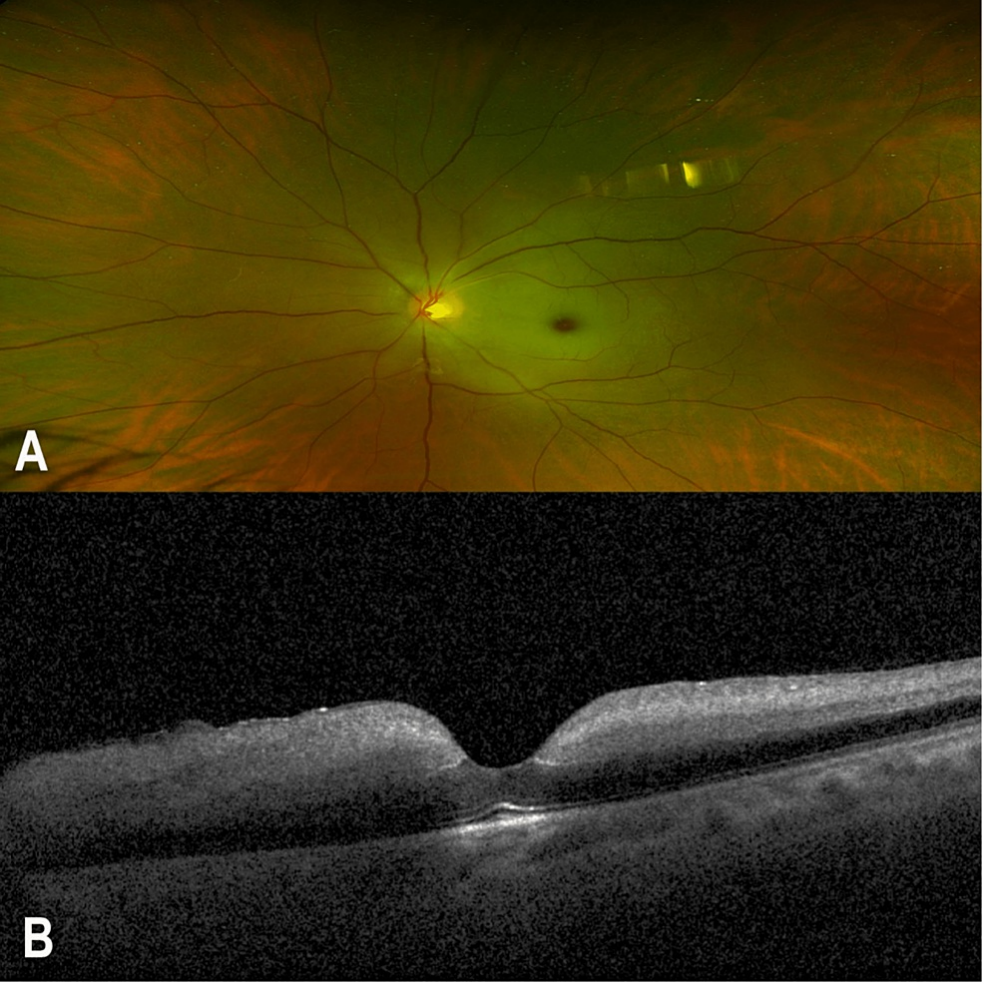
Multimodal imaging of the left eye of a 33-year-old male with sickle cell trait. (A) A colored fundus photo of the left eye showing a cherry-red spot at the macula. (B) A spectral-domain optical coherence tomography showing inner retinal thickening with poor differentiation of retinal layers.

A 33-year-old male with no previous medical history presented to the ophthalmological emergency room complaining of a sudden onset of painless and profound loss of vision in his left eye for around 12 hours. On taking a history, the patient denied taking any medication use, past eye trauma, or surgery. On detailed ophthalmologic examination, the best-corrected visual acuity (BCVA) was 20/20 in the right eye and hand movement in the left eye. There was an afferent pupillary defect on the left pupil. The slit lamp examination showed that the right eyelids, conjunctiva, and cornea were within normal limits. The anterior chamber was deep and quiet. The lens was clear. Dilated fundus examination of the right eye showed a flat retina, normal macular reflex, healthy optic nerve head, and normal retinal vasculature. The left eye examination showed lids, conjunctiva, and cornea within normal limits. The anterior chamber was deep and quiet. The lens was clear. Dilated fundus examination of the left eye showed a picture of CRAO with pale whitening and swelling of the retina with a cherry-red spot in the macula. Optical coherence tomography (OCT) of the left eye showed marked edematous and thickened inner retina with poorly differentiated retinal layers (Figure 1).

**Figure 2:**
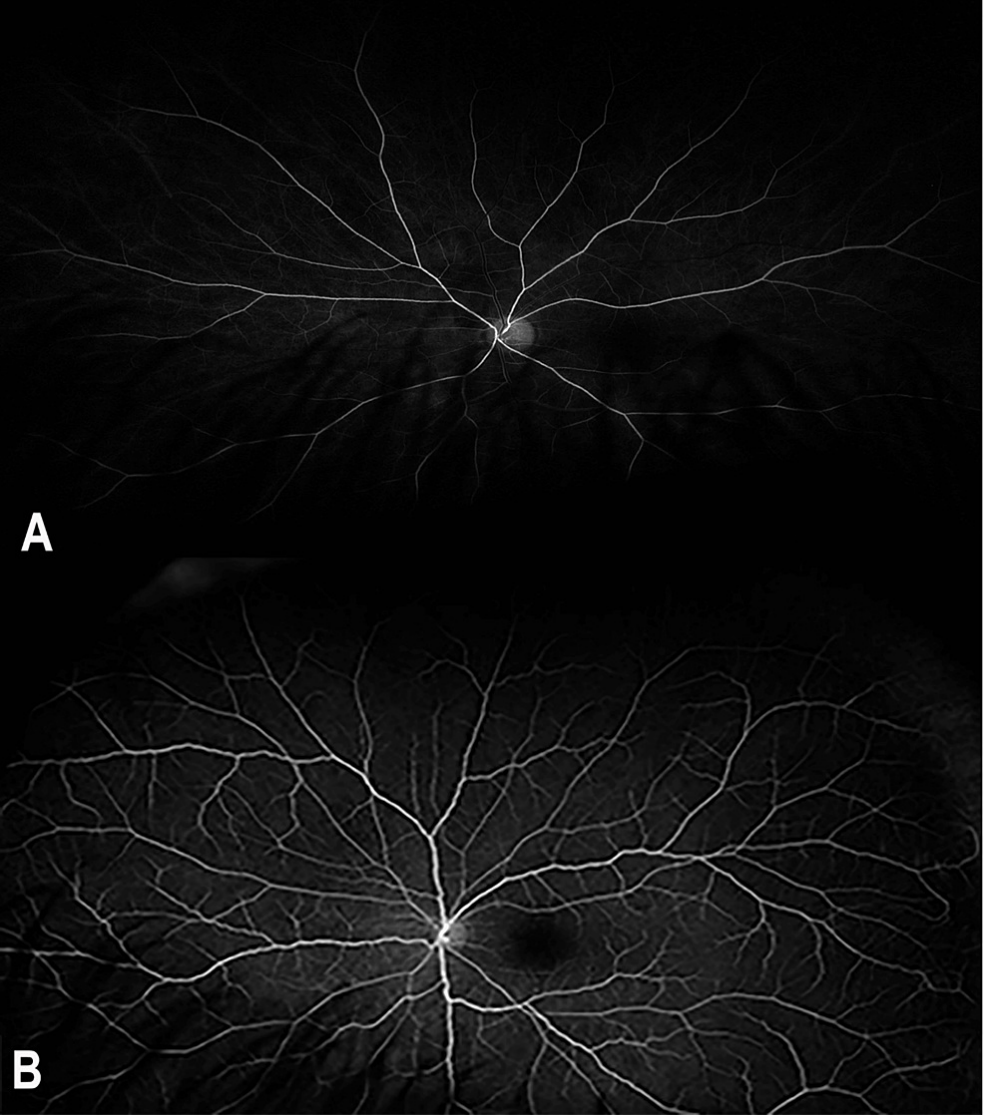
FFA of the left eye upon presentation. (A) An early phase FFA shows a delay in the filling of retinal arteries. (B) A late-phase FFA shows reduced macular perfusion. FFA, fundus fluorescein angiography

Fundus fluorescein angiography showed an early delay of the arterial filling with persistently reduced macular perfusion (Figure 2).

A complete workup was performed, and all the results came back negative except hemoglobin electrophoresis, which showed sickle cell trait (SCT); hemoglobin A1C was 7.8%. The patient also had elevated triglycerides. The basic workup lab results are shown in Table 1.

**Table 1:** The patient's workup lab results

Test item	Value	Reference range
White blood cell count	8.3 x 10^9^/L	4.5-11.0 x 10^9^/L
Neutrophil %	52.0%	54%-62%
Lymphocyte %	39.8%	25%-33%
Monocyte %	5.6%	3%-7%
Eosinophil %	1.1%	1%-3%
Basophil %	1.4%	0%-0.75%
Red blood cell count	6.57 x 10^12^/L	Male: 4.3-5.9 x 10^12^/L
Hemoglobin	113 g/L	Male: 135-175 g/L
Hematocrit	0.404 L/L	Male: 0.41-0.53 L/L
Mean corpuscular volume	61.5 fL	80-100 fL
Mean corpuscular hemoglobin	17.2 pg/cell	25-35 pg/cell
Mean corpuscular hemoglobin concentration	279 g/L	320-360 g/L
Red cell distribution width	17.3%	12%-15%
Platelet count	328 x 10^9^/L	150-400 x 10^9^/L
Mean platelet volume	8.3 fL	7.5-11.5 fL
Sickle cell screen	Positive	-
Hemoglobin A	68.60	-
Hemoglobin F	1.6	-
Hemoglobin S	28.6	-
Hemoglobin A2	1.2	-
Diagnosis	Sickle cell trait (heterozygous A/S) expected range: hemoglobin A levels are higher than hemoglobin S	-
C-reactive protein	2.16 mg/L	<3.0 mg/L
Glucose – random	11.2 mmol/L	<7.77 mmol/L
Prothrombin time	11.0 seconds	11-15 seconds
International normalized ratio	1.1	1.1 or below
Activated partial thromboplastin time	22 seconds	25-40 seconds
Is the patient receiving anticoagulants?	No	-
Glycosylated hemoglobin (HbA1C)	7.8%	<5.7%
Cholesterol – total	4.9 mmol/L	<5.2 mmol/L
Cholesterol (low-density lipoprotein)	1.00 mmol/L	1.0-1.6 mmol/L
Cholesterol (high-density lipoprotein)	3.0 mmol/L	<4.2 mmol/L
Triglycerides	4.39 mmol/L	<1.70 mmol/L

**Figure 3:**
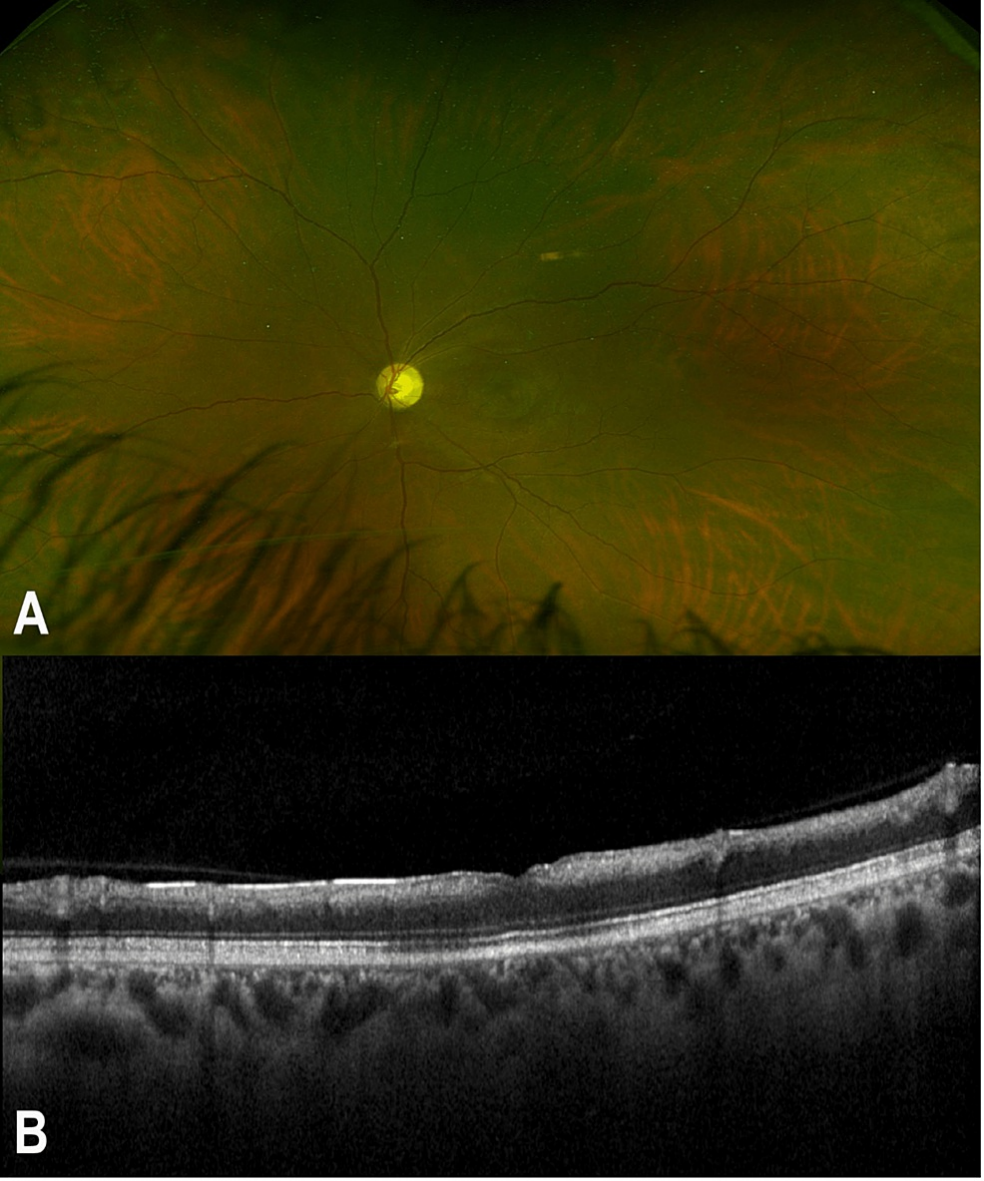
Multimodal imaging of the left eye two months later. (A) A colored fundus photo shows an atrophic macular area with a pale optic disc. (B) An optical coherence tomography shows full-thickness retinal thinning and atrophy.

A more intensive workup was performed, including the levels of lupus anticoagulant, anticardiolipin, factor V Leiden, antithrombin, protein C, and protein S, which were all unremarkable. Electrocardiogram, transthoracic echocardiography, and carotid Doppler were all within normal limits. Then, the patient was referred to internal medicine and hematology consultants for further management. Two months later, visual acuity was reduced to light perception, and atrophic macular changes with the pale optic disc were noted, as well as full-thickness atrophy of the retina and reverse shadowing on OCT (Figure 3).

## Discussion

Sickle cell disease is a group of inherited red blood cell diseases in which an amino acid substitution in hemoglobin protein leads to abnormal hemoglobin structure and function. In SCT, patients usually have one normal gene for normal hemoglobin, whereas the other gene codes for abnormal sickling hemoglobin; it is estimated that the prevalence rate of SCT in the United States is around 9% among African Americans [9]. In the past, SCT was considered a benign condition because the patients did not get a vaso-occlusive crisis. Compared with the general population, SCT patients usually have the same mortality rate and the same quality of life [10]. Many ophthalmologists also consider the trait status of the patients as a benign entity [11]. However, recent studies have shown that SCT is probably linked to complicated hyphema, acute chest syndrome, and venous thromboembolic events [12]. In addition, CRAO has been reported in many subtypes of sickle cell hemoglobinopathies such as SS and S-thal, but it is rare in SCT [13].

This case reports spontaneous CRAO in an otherwise healthy young male and suggests that CRAO in these cases usually has more obscure and diverse etiological causes that require extensive workup. Concurrence of undiagnosed diabetes mellitus (DM) and SCT makes these presentations overlap, resulting in difficulty in establishing which causes the CRAO and which is a trigger factor. However, most likely undiagnosed type 2 DM may be the trigger but not the cause of CRAO, as uncontrolled DM can lead to oxidative stress and dehydration, leading to the environmental favor of sickling. Even more, there are reports in the literature of spontaneous sickling leading to CRAO in patients with SCT [14-16]. These patients need close follow-up and observation for any CRAO complication such as the risk of development of ocular neovascularization, as well as a proper referral to a medical doctor to manage all risk factors such as control of type 2 DM. This case gives us an example of an undiagnosed systemic disease that may be picked up and diagnosed by an ophthalmologist.

## Conclusions

In young individuals, CRAO is frequently due to more complex etiological factors requiring extensive systemic workup. In addition, the association of the SCT with early DM may have contributed to the development of CRAO. Patients with spontaneous CRAO need to be closely monitored and watched for any CRAO complications. Referral to a medical doctor should also be made to handle all risk factors, especially DM. This case illustrates how an ophthalmologist might identify and diagnose a systemic condition that has gone undetected.
